# Impact of Short Message Service (SMS) Education Based on a Health Promotion Model on the Physical Activity of Patients with Type II Diabetes

**DOI:** 10.21315/mjms2018.25.3.7

**Published:** 2018-06-28

**Authors:** Hamideh Lari, Azita Noroozi, Rahim Tahmasebi

**Affiliations:** 1 Department of Health, Bushehr University of Medical Sciences, Bushehr, Iran; 2 The Persian Gulf Marine Biotechnology Research Center, Bushehr University of Medical Sciences, Bushehr, Iran; 3 Department of Biostatistics, Bushehr University of Medical Sciences, Bushehr, Iran

**Keywords:** health promotion model, physical activity, diabetes type II, short message service, mHealth

## Abstract

**Introduction:**

Physical activity is the most important self-management behaviour in diabetes. The aim of this study was to evaluate the impact of a short message service (SMS) based on a health promotion model (HPM) on the physical activity of diabetic patients.

**Methods:**

This quasi-experimental study consisted of 37 type II diabetes patients in an SMS group and 36 type II diabetes patients in a control group. The patients in both groups completed written consent forms and questionnaires at the beginning of the study. The patients in the SMS group received training messages within two weeks (two or three messages daily) in the field of physical activity based on HPM constructs. Both groups completed questionnaires in three stages (at the beginning of the study, four weeks after the first visit, and three months later) comprising demographic factors, questions regarding the constructs, and 7-day physical activity recall. After data collection, statistical analysis was conducted using an independent *t*-test, a Chi-square test, and a repeated measures analysis of variance (ANOVA).

**Results:**

As compared with the control group, changes in mean scores of perceived self-efficacy (*P* = 0.001) and family support (*P* = 0.046) of physical activity in the training group were significantly greater and perceived barriers (*P* < 0.001) were significantly lower over time. The physical activity performance of the SMS group was better three months after training as compared with that of the control group (*P* < 0.001).

**Conclusion:**

The results demonstrated the efficacy of training messages in changing the beliefs and physical activity behaviours of diabetic patients.

## Introduction

Diabetes is increasing worldwide. Due to aging, urbanisation, an increased prevalence of obesity, and physical inactivity, the global burden of diabetes type II is increasing (1). Diabetes affects over 180 million people (2). According to estimates, the numbers of patients with diabetes will double by 2030, with about half the diabetes population being in Oceania and Asia (2). By 2025, there will be a predicted 122% increase in the number of adults with type II diabetes (3).

In addition to the high prevalence of this disorder, the cost of diabetes is significant. In many countries, at least 10% of the total cost of health care is spent on diabetes (4). Diabetes control depends on self-management behaviours, including nutrition control, sufficient physical activity, adherence to medication, and blood sugar monitoring to avoid serious complications and mortality (5). Despite the importance of physical activity in diabetes control, in the U.K., 68% of type II diabetic patients and 61% of patients with type I diabetes have been classified as inactive and sedentary (6). The American Diabetes Association recommends that patients with type II diabetes should have at least 150 min of aerobic activity of moderate intensity each week or 90 min of intensive aerobic exercise each week (7).

Monitoring of physical activity is important in diabetic patients, and this activity can be monitored via a training intervention (8). However, the impact of any training depends on appropriate application of behavioural theories and appropriate training methods (8). The basis of diabetes control is self-management. The main emphasis of health promotion models (HPMs) is self-regulation (i.e., using internal standards and self-assessment as a means of motivation, adjustment of behaviour, and adjustment of the external environment). Thus, HPMs seem to be effective for inducing behavioural change in diabetic patients. The revised model includes the following concepts of health-promoting behaviour: i) individual characteristics and experiences, ii) behaviour-specific cognition and affects, and iii) behavioural outcomes. The concept of individual characteristics and experiences includes personal factors and prior related behaviours. The concept of behaviour-specific cognition and affects includes constructs, such as perceived benefits and barriers, perceived self-efficacy, activity-related affect, interpersonal influences, and situational influences. Pender et al. tested their HPM in multiple studies and identified constructs, such as personal factors (perceived health status), perceived benefits and barriers, perceived self-efficacy, and interpersonal influences (social support) as the best predictors of a health-promoting lifestyle (9).

Researchers have used a variety of technologies to provide interventions in health promotion, with the cell phone proving to be an effective technology in all aspects of human life (10). Cell phone penetration in Iran today has reached 90% (10). Cell phones and short message services (SMS) are modern means of communication and interaction. They have a wide range of functionalities and features, such as high-speed, permanent access, cost effectiveness, relative security, storage capability, flexibility, and content attractiveness (11, 12). As most people today have busy lives, and traveling and attending training classes are not easy, SMS may be a suitable method to deliver training interventions. Previous studies demonstrated that sending SMS was an effective way to promote self-management behaviours in diabetic patients (12, 13). However, in these studies, the training messages were not based on a HPM.

Considering the importance of physical activity in diabetics and the pervasiveness of the cell phone as a communication tool, this study was designed to determine the impact of a distant training program delivered through SMS and based on a HPM on the physical activity of patients with type II diabetes. The results can be used to design a training program for diabetic patients at the community level.

## Material and Methods

### Study Design

This clinical trial study was carried out from October 2016 to February 2017 to assess the impact of training using SMS on the physical activity of patients with type II diabetes who were referred to two diabetes clinics in the city of Bushehr, a southwestern province in Iran.

The inclusion criteria were the ability to read and write, having no diabetic foot ulcers, a willingness to participate in the study, and having diabetes for one or more years. The exclusion criteria included functional inability to walk without a cane, inability to walk one mile without a rest, inability to continue participation for at least three months, and having cardiovascular disease.

Based on a similar study (14), the sample size was estimated as 36 subjects for each group, considering attrition rates (10%) in a 3-month follow up. Thus, about 40 subjects were required for each group (an SMS group and a control group). Two diabetes clinics were selected for sampling. Based on a review of 1,775 records of patients in Haftom-e-Tir Diabetes Clinic and 514 records in a diabetes clinic of the Social Security Hospital, taking into account the inclusion criteria, 145 patients in Haftom-e-Tir Clinic and 89 patients in the Social Security Hospital were deemed eligible. Eighty of the 234 patients were selected by assigning a code to each eligible person and random selection using Excel software. Potential participants were contacted by telephone. After identification of subjects willing to participate, the individuals were randomly divided into two groups (training with SMS or a control) by lottery. Of the 40 participants in each group, three patients in the SMS group and four patients in the control group were unable to continue the study and were excluded. Ultimately, 37 patients in the SMS group and 36 patients in the control group continued the study.

### Method and Data Collection

At the beginning of the study, the patients in both groups attended the clinic on a particular day determined by themselves and completed a written consent form and then questionnaires. These included a demographic questionnaire, HPM constructs, and 7-day physical activity recall questionnaire. The appointment interval was one hour per day to allow the researchers sufficient time to administer the physical activity questionnaire through interviews and to monitor the questionnaires completion.

The patients in the SMS group received training messages based on a HPM. The training consisted of two or three messages per day for two weeks from Saturday to Thursday for 12 days. The composition of the messages was as follows:

Day 1: Training about the appropriate amount of physical activity to control blood sugar and the impact of physical activity on diabetes (increased perceived benefits).
Day 2: Physical and mental benefits of physical activity in patients with diabetes (increased perceived benefits).
Days 3 and 4: Sports rules and some tips about hiking to change physical activity step by step and gradually increase this activity over a 3-month period (increased perceived self-efficacy).
Days 5 and 6: Observing specific rules before physical activity in the field of blood sugar control and not consuming snacks before undertaking physical activity (reduced perceived barriers).
Days 7 and 8: Undertaking appropriate physical activity based on blood sugar and symptoms of low blood sugar and taking appropriate measures to eliminate it (reduced perceived barriers).
Day 9: Complications of diabetes and appropriate physical activity (increased perceived benefits).
Day 10: Increasing the ability to perform physical activity or enhancing self-efficacy by, for example, emphasising a gradual increase in physical activity in accordance with the person’s ability and modifying the person’s views on the usefulness of an increase in heart rate associated with physical activity (increased perceived self-efficacy).
Day 11: Identifying barriers to physical activity, including problems related to physical activity during periods of high blood sugar, and appropriate measures to eliminate barriers, such as training to reduce muscle cramps after an initial increase in physical activity (reduced perceived barriers).
Day 12: Asking support from friends and family in care and accompaniment (perceived support). In addition, to enhance perceived support, a contact number of an active member of the person’s family or the number of an active friend was taken to send a message. In addition to the benefits, barriers, and self-efficacy, recommendations on care, acceptance, trust, and accompanying the patient were sent to these contacts, and they were asked to support the diabetic patients in terms of physical activity.

On Friday, the participants in the SMS group put questions to the researcher and received the answers. In addition, after two weeks of daily training messages, the participants in the SMS group received two messages weekly (repetition of the initial messages based on the HPM constructs) for up to three months. When the message was read by the individual in the SMS group, a confirmation message was sent to the researcher from the patient.

In the first stage, four weeks after questionnaire completion, the participants in both groups completed the questionnaires based on the HPM constructs. In addition, three months after data collection in the second stage, both groups completed a questionnaire related to these constructs and a 7-day physical activity recall questionnaire.

At the beginning of the study, after giving written consent, the participants in the control group completed the questionnaires on demographics, HPM constructs, and 7-day physical activity recall. Four weeks later, they completed questionnaires based on the HPM constructs. Three months later, they completed the questionnaires based on the HPM constructs and 7-day physical activity recall again. At the end of the study, they did not receive training materials.

### Instruments and Measures

The data collection tool in this study was a three-part questionnaire. The first part contained information about the participant’s characteristics (e.g., age, sex, education, household income, body mass index [BMI], type of medication, prior related behaviours, and perceived health status) and experiences.

Perceived health status was determined by a 12-item short form health survey examining physical and mental health. Montazeri et al. reported Cronbach’s alpha of 0.73 (physical health aspect) and 0.72 (mental health aspect) for this instrument (15). In this study, Cronbach’s alpha coefficient was 0.79 for all the tools.

The second part of the questionnaire contained questions related to the HPM constructs.

#### Perceived benefits

Agreement or disagreement with the benefits of physical activity were examined by 28 questions, using a 4-point Likert scale designed by Sechrist et al. (16). Cronbach’s alpha coefficient of the scale was reported to be 0.89 (16). In this study, Cronbach’s alpha coefficient was 0.93.

#### Perceived barriers

Sechrist et al.’s (16) 14-point Likert scale was used to assess participants’ perceptions of barriers to physical activity. Cronbach’s alpha coefficient of the scale was reported to be 0.77 (16). In this study, it was 0.74.

#### Perceived social support

The participants’ perceptions of support from family and friends in the field of physical activity was measured by 15 and 5 questions, respectively (a total of 20 questions), with a 5-point Likert scale. Cronbach’s alpha coefficients were reported to be 0.9 and 0.86 for support from family and friends, respectively (17). In this study, it was 0.94 for all the tools.

#### Self-efficacy

The questionnaire developed by Noroozi et al. (18) was used to examine the confidence of the participants in undertaking regular physical activity under different conditions. This questionnaire has 18 questions, which are scored on a percentage scale (0 to 100%). Cronbach’s alpha coefficient of the scale was 0.92 (18). In this study, it was 0.92.

The third part of the questionnaire measured physical activity based on 7-day physical activity recall. The questionnaire was completed during a semi-structured interview. In the interview, the subjects were asked to list the activities they had undertaken in the last seven days, starting with the day before and then going backwards. They were asked to determine the duration (in minutes), intensity (based on changes in heart rate as compared with walking and running), and type of each activity (daily activities or leisure activities). Using the instructions given in these tools, the metabolic equivalent (MET) was calculated for the last week. Previous research demonstrated the reliability of this questionnaire, reporting an intraclass coefficient range between 0.34 and 0.99. Research also showed that this questionnaire was a useful tool to assess the amount of physical activity (19).

**Figure 1:**
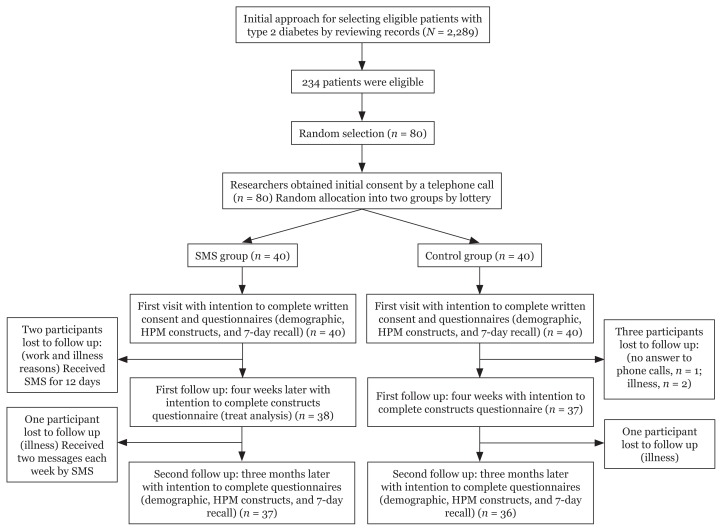
CONSORT flow chart of the participants

The heights and weights of the participants were measured to determine the METs and BMIs. The data were analysed by the Statistical Package for Social Sciences Software (SPSS), version 22.0. Descriptive statistics, a Chi-square test, an independent t-test, and a repeated measures analysis of variance (ANOVA) were used for data analysis. A CONSORT flow diagram of the study is displayed in Figure 1.

## Results

In total, 73 diabetic patients (SMS group, *n =* 37; control group, *n* = 36) completed the study. There were no significant differences between the SMS and control groups in individual characteristics and experience (demographic factors). For instance, the average age of the participants in the SMS group was 46.10 (9.14) years, and the average age in the control group was 49.13 (9.07) years (*P* = 0.160). The mean (SD) BMI in the SMS group and control group was 27.06 (5.19) and 26.92 (3.93), respectively (*P* = 0.897), and the duration of diabetes in the SMS group and control group was 7.73 (7.18) and 8.78 (5.74), respectively (*P* = 0.493). Other demographic characteristics are shown in Table 1.

The participants in the SMS and control groups were also similar as regards their health beliefs related to physical activity before education, but the difference between the two groups was statistically significant after training in several constructs (Table 2).

Comparison of the pre- and post-test results (immediate and three months later) of the SMS group by a repeated measures ANOVA revealed changes in perceived health status, perceived self-efficacy, perceived barriers, and family support, with an increase in perceived health status, self-efficacy, and family support (*P* < 0.001), as well as a decrease in perceived barriers (*P* < 0.001). There were no statistically significant changes in the constructs on perceived benefits and support from friends (*P* > 0.05). In contrast, in the control group, there were no significant changes between the pre- and post-test scores for any of the constructs.

**Figure 2:**
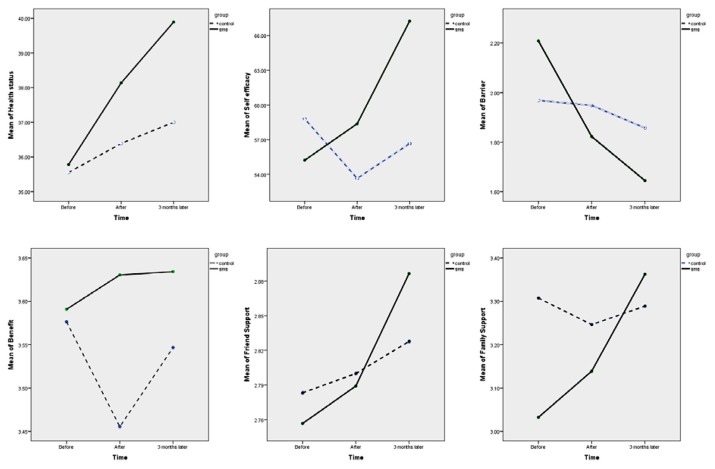
Comparison of constructs’ scores change in over time between groups

Comparison of the two groups over time revealed no between-group difference in scores for perceived health status, perceived benefits, and support from friends (*P* > 0.05). However, self-efficacy and family support were perceived to be higher and barriers were perceived to be lower in the SMS group as compared with the control group (Table 2). A comparison of changes in the constructs’ scores between the SMS and control groups is presented in Figure 2.

Pre- and post-test mean differences in the MET between the two groups were statistically significant (*P* < 0.001). The mean (SD) of the MET in the SMS and control groups over time is shown in Table 3.

## Discussion

This study showed that SMS training messages improved perceptions of self-efficacy and family support and reduced barriers to physical activity among diabetic patients, subsequently increasing their level of physical activity. However, short messages were not effective in improving perceived benefits of physical activity or support of friends for physical activity.

Several studies reported that self-efficacy was a determinant of physical activity behaviour (20–22). Thus, its promotion is important in encouraging physical activity. Various studies that used different training methods, including individual training (23, 24), group training (25), and training by SMS (26), indicated increased self-efficacy in physical activity, confirming the findings of a recent study. Accordingly, providing positive feedback and recording daily progress were effective in strengthening self-efficacy in physical activity. However, one study found that SMS failed to increase self-efficacy in self-management behaviours in diabetic patients (13). This study attributed the failure to multiple self-management behaviours, with physical activity being only one component of these behaviours. Therefore, SMS may be ineffective in promoting components of self-management behaviours other than physical activity, indicating the need for more research on the efficacy of SMS on each component of self-management behaviour.

In the present study, SMS reduced the participants’ perceptions of barriers to undertaking physical activity. Several studies that provided individual or group training confirmed this finding (13, 23, 25). However, training provided through printing materials did not change people’s perception of barriers to undertaking physical activity (27). This finding indicates that people may need to be trained interactively to change their perceptions of barriers to behaviours. Due to its simplicity and effectiveness, SMS-based training may be a good way of reducing perceived barriers.

In patients with specific conditions and diseases, family support is very important in changing health behaviours to promote health (28). In this study, sending training messages through SMS to the families of diabetic patients improved the level of familial support and the participants’ perceptions of social support by family members in the field of physical activity. Studies where training was conducted in the presence of family members of patients achieved similar results (23, 29). Researchers believe that if a person has a positive attitude to a behaviour, and people who are important to them confirm this behaviour, the person performs the behaviour (30). As sending short messages to friends failed to increase the participants’ perceptions of friends’ support in the field of physical activity, it seems that family members are more important for diabetics. Thus, sending short messages to family members seems to be more effective than sending them to friends.

In this study, sending training messages failed to change the participants’ perceptions of the benefits of physical activity, contradicting the findings of several studies (2, 23, 25). The discord could be due to the high level of perception of the benefits of physical activity at the beginning of the study. Therefore, the training messages failed to change their perceptions.

As several factors, including age, social class, education level, and economic status (31), influence people’s perceptions of health, it should be expected that training interventions aimed at changing behaviour do not have much impact on health status. In this study, sending training messages did not change the participations’ perceptions of their health status.

This study showed that changing health beliefs about self-efficacy, family support, and barriers to physical activity significantly increased the mean MET, in other words, the duration of physical activity in the SMS group. Several studies that incorporated a health promotion program demonstrated the impact of the program on physical activity promotion (23, 25, 32). In addition, in some studies, sending SMS without taking into account the theoretical framework was effective in increasing physical activity (11, 14, 33). All these studies confirmed the recent findings. In the present study, the theoretical framework applied was effective in changing diabetic patients’ beliefs. In this study, more persistence and continuity are expected from this behaviour, confirming the need for more studies in this field. Therefore, the delivery of SMS messages based on a HPM may be a useful approach for improving diabetic patients’ beliefs about physical activity and promoting physical activity in these patients.

## Conclusion

According to the results, it seems that the design and implementation of a training program using a HPM had positive impacts as regards changes in health beliefs and subsequently, promotion of physical activity. Therefore, we recommend the use of cell phones and SMS as readily available, affordable tools to change the health beliefs of diabetic patients and to induce health-promoting behaviours, such as physical activity.

## Figures and Tables

**Table 1:** Demographic characteristics in two groups prior to training

Demographic variables		SMS group		Control group		Chi-square statistics	*P*-value
	
		*n*	%	*n*	%		
Gender	Female	17	45.9	17	47.2	0.913	0.550
	Male	20	54.1	19	52.8		
Education level	Diploma	23	62.2	20	55.6	0.596	0.742
	Academic education	14	37.8	16	44.4		
Married status	Married	32	86.5	33	91.7	0.502	0.371
	single	5	13.5	3	8.3		
Job	Housekeeper	15	40.5	9	25	8.152	0.227
	Employee	14	37.8	9	25		
	pensionary	8	21.7	18	50		
Drug type	Metformin	10	27	9	25	1.561	0.668
	Insulin	3	8.1	5	13.9		
	Combine	24	64.9	22	61.6		

**Table 2:** Constructs’ scores of health promotion model during intervention

Constructs	Time	SMS group	Control group	F (df_1_, df_2_)	b *P*-value
	
		Mean (SD)	Mean (SD)		
Perceived health status	Before education	35.78 (6.62)	35.55 (5.95)	1.98 (2, 142)	0.142
	After education	38.13 (5.87)	36.38 (5.87)		
	3 months later	39.89 (5.60)	37.00 (6.20)		
F(df_1_, df_2_)		9.01 (2, 72)	1.23 (2, 70)		
a *P*-value		0.001	0.299		

Perceived Self-efficacy	Before education	55.21 (21.35)	58.80 (22.54)	7.03 (2, 142)	0.001
	After education	58.37 (20.16)	53.65 (20.10)		
	3 months later	67.25 (13.57)	56.65 (21.33)		
F(df_1_, df_2_)		9.25 (2, 72)	2.24 (2, 70)		
a *P*-value		*P* < 0.001	0.114		

Perceived barrier	Before education	2.20 (0.40)	1.96 (0.49)	10.52 (2, 142)	< 0.001
	After education	1.82 (0.43)	1.94 (0.38)		
	3 months later	1.64 (0.31)	1.85 (0.36)		
F(df_1_, df_2_)		28.20 (2, 72)	1.39 (2, 70)		
a *P*-value		*P* < 0.001	0.256		

Perceived benefit	Before education	3.59 (0.32)	3.57 (0.35)	2.08 (2, 142)	0.129
	After education	3.63 (0.34)	3.45 (0.39)		
	3 months later	3.63 (0.28)	3.54 (0.31)		
F(df_1_, df_2_)		0.39 (2, 72)	0.14 (2, 70)		
a *P*-value		0.676	0.097		

Friend support	Before education	2.75 (1.23)	2.78 (1.05)	0.07 (2, 142)	0.931
	After education	2.78 (0.98)	2.80 (0.97)		
	3 months later	2.88 (1.16)	2.82 (1.02)		
F(df_1_, df_2_)		0.34 (2, 72)	0.03 (2, 70)		
a *P*-value		0.714	0.968		

Family support	Before education	3.03 (0.73)	3.30 (0.82)	3.14 (2, 142)	0.046
	After education	3.13 (0.72)	3.24 (0.69)		
	3 months later	3.36 (0.76)	3.28 (0.76)		
F(df_1_, df_2_)		1.05 (2, 72)	0.04 (2, 70)		
a *P*-value		0.002	0.838		

a Comparison of mean score changes over time in each group

b Comparison of changes in mean scores over time between groups

**Table 3:** Mean metabolic equivalent of task (MET) in two groups during intervention

Group	Before education Mean (SD)	Three months later Mean (SD)	Mean differences	95% CI of Diff.	*T*-value	*P*-value
Control	2546.98 (479.48)	2500.55 (423.08)	−46.43	(−93.03, 0.17)	−2.02	0.051
SMS	2554.46 (495.34)	2634.71 (502.01)	80.25	(46.22, 114.28)	4.78	< 0.001
